# The Biotechnological Potential of the Marine Diatom *Skeletonema dohrnii* to the Elevated Temperature and *p*CO_2_

**DOI:** 10.3390/md18050259

**Published:** 2020-05-15

**Authors:** Satheeswaran Thangaraj, Jun Sun

**Affiliations:** 1 Research Centre for Indian Ocean Ecosystem, Tianjin University of Science and Technology, Tianjin 300457, China; satheeswaran1990@gmail.com; 2 Tianjin Key Laboratory of Marine Resources and Chemistry, Tianjin University of Science and Technology, Tianjin 300457, China; 3 College of Food Engineering, Tianjin University of Science and Technology, Tianjin 300457, China

**Keywords:** diatom, algae, *p*CO_2_, temperature, gene expression, lipids, fatty acid, biofixation, biotechnological applications, industrial applications

## Abstract

Marine diatoms are promising candidates for biotechnological applications, since they contain high-value compounds, naturally. To facilitate the production of these compounds, stress conditions are often preferable; however, challenges remain with respect to maximizing a metabolic potential for the large-scale cultivation. Here, we sequenced the transcriptome of diatom *Skeletonema dohrnii* under the actual (21 °C, 400 ppm) and elevated (25 °C, 1000 ppm) temperature and *p*CO_2_ condition. Results indicated that cells grown at higher temperature and *p*CO_2_ showed increasing growth rate, pigment composition, and biochemical productivity as did the expression of chlorophyll, carotenoid and bioactive compound related genes or transcripts. Furthermore, performing de novo transcriptome, we identified 32,884 transcript clusters and found 10,974 of them were differentially expressed between these two conditions. Analyzing the functions of differentially expressed transcripts, we found many of them involved in core metabolic and biosynthesis pathways, including chlorophyll metabolism, carotenoid, phenylpropanoid, phenylalanine and tyrosine, and flavonoid biosynthesis was upregulated. Moreover, we here demonstrated that utilizing a unique bio-fixation ability, *S. dohrnii* is capable of suppressing central carbon metabolism to promote lipid productivity, fatty acid contents and other bioactive compounds under high temperature and *p*CO_2_ treatment. Our study suggests that this *S. dohrnii* species could be a potential candidate for wide-scale biotechnological applications under elevated temperature and CO_2_ conditions.

## 1. Introduction

In recent years, an increasing consideration has been devoted to the possibility of growing microalgal culture for commercial purposes. These interests are due to microalgal natural sources for wide-scale biotechnological applications including human food [1], animal feed [2], cosmetics [3], drugs [4], health products [5], biodiesel [6], fertilizers [7] and wastewater treatment [8]. On the other hand, increasing anthropogenic CO_2_ is causing major changes on our earth. To mitigate the anthropogenic CO_2_ concentrations with alternative renewable energy and other valuable products [9], scientists are focusing on the choice of cultivating microalgal strain, which could tolerate higher CO_2_ and simultaneously produce bioactive compounds and natural products [10,11].

Marine diatoms are highly diverse and significant contributors to global primary productivity; they are also considered as attractive organisms for the combined CO_2_ biofixation and natural products [12,13]. Recently, many physiological investigations evaluated diatoms’ natural product efficiency under stress conditions, including CO_2_ [6], temperature [14] nutrients [15] and pH [16]. These investigations revealed that diatoms tend to increase their bioactive compounds and natural products under stressful conditions. Despite this, the metabolic potential of biosynthesis pathways is not fully explored for wide-scale biotechnological applications. Recent transcriptome investigation on *Thalassiosira rotula* showed that silica stress leads to an increase in the expression of secondary metabolites production [17], while phosphorus and nitrogen deficiency on *T. rotula* revelated transcriptome remodeling to increase the lipid accumulation [18]. In addition, with the nutrient’s deprivation, light intensity also reported remodeling the metabolites to increase the lipid accumulation [19]. Although a similar metabolic adjustment may occur by elevated temperature and *p*CO_2_ stress conditions on diatoms, it is still not explored the altered biosynthesis pathways’ response to combining temperature and CO_2_ variation. Moreover, to date, most of the investigations focus on fewer well-known diatoms for commercial purposes; therefore, it is important to disclose the new metabolic profile of new species, which could be a valuable alga for future industrial applications.

Among diatoms, *Skeletonema* is commonly found in marine and coastal environments and it is considered as an important group in diatom research because of its similar physiology to other species, global abundance and is easy to maintain in laboratory. Among them, *Skeletonema dohrnii* is a cosmopolitan red tide bloom forming species, widely distributed in temperature regions [20]. In our previous proteomic study between Si-deplete and replete conditions, we demonstrated that *S. dohrnii* could show significant differences at proteomic level like other model diatoms when changing environmental conditions [20]. However, unlike other species, this species has not been explored using omics approaches for the industrial application, and to date, the transcriptome profile of this species has not been explored and there are no relevant genomic data existing to understand this species biosynthesis mechanism under any stress conditions [20]. High-throughput sequencing helps us to explore non-model species to discover new physiological pathways, and bioactive compounds [21] with simple sequence repeats (SSRs microsatellites) for other evolution studies.

Here, we utilize the de novo transcriptome sequencing method (i) to investigate the metabolic potential of the diatom *S. dohrnii* as a promising alga for wide-scale biotechnological applications under elevating temperature and *p*CO_2_ conditions (ii) and also to understand the diatom’s biosynthesis mechanistic regulation of pathways related to bioactive compounds and natural products under stressful conditions.

## 2. Results

### 2.1. Physiological and Biochemical Response

We first analyzed the growth rate of *S. dohrnii* grown at LC (low carbon and low temperature) and HC (high carbon and high temperature) conditions (Table 1). Result shows that cells grown at HC conditions reveal considerably higher growth rate (1.23 ± 0.15 day^−1^) compared with cells grown in the LC environment (0.76 ± 0.05 day^−1^). Consistently, we also found cell density was higher (*p* < 0.01) at HC conditions 297.3 ± 9.71 (cells mL^−1^) than cells treated with LC conditions 233.3 ± 9.07 (cells mL^−1^) (Table 1). The photosynthetic pigments of Chl-*a* and carotenoid indicating extensive variations between each treatment (*p* < 0.05), showing decreased quantity under LC and increased quantity under HC environment (Table 1). In terms of protein synthesis, *S. dohrnii* cells grown under high and low CO_2_ concentrations show notable differences by increased protein synthesis 3.7 ± 0.1 (pg cell^−1^) at HC and decreased 3.1 ± 0.2 (pg cell^−1^) in the LC treatments (Table 1). The high temperature and *p*CO_2_ concentration also interacted with the carbohydrate content, showing an increased carbohydrate accumulation, compared with low temperature and CO_2_ concentration (Table 1), however, it shows insignificant variations (*p* > 0.05) between the treatments. The lipid content and productivity exhibit substantial differences (*p* < 0.01) between LC and HC conditions, suggesting elevation in temperature and CO_2_ remarkably induced lipid biosynthesis and accumulation (Table 1). Furthermore, the results obtained from seawater carbonate chemistry at both HC and LC conditions are summarized in Table 2. Results show that cells treated with HC condition notably increased Dissolved Inorganic Carbon *(*DIC), but decreased pH and CO_3_^−^^2^.

### 2.2. RNA Sequencing, de Novo Assembly, and Functional Annotation

The transcriptome of six samples of *S. dohrnii* grown at LC and HC conditions were sequenced using the Illumina High-Throughput sequencing platform. The Illumina reads ranging from 75.37 to 77.13 million/sample (on average 76.54 million reads) were obtained in this study (Table S1). Of each sample, the clean data reached above 10 Gb; the Q30 base percentages were 89% or more. The clean data of six samples of low and high carbon grown cells were de novo assembled using Trinity software and finalizes 32,884 transcripts with a mean length of 1816 bp, a N50 of 2810 bp and N90 of 957 bp (Table S1).

We annotate the assembled unigenes into NCBI-non-redundant proteins (NR), nucleotide (NT), Swiss-Prot, Kyoto Encyclopedia of Genes and Genomes (KEGG), Eukaryotic Orthologous Groups (KOG), Pfam protein data base, and Gene Ontology (GO). Among the achieved 32,884 unigenes, 22,261 (67.70%), 2960 (9.00%) and 11,583 (35.19%) have matched with the NR, NT, and Swiss-Prot databases respectively; 13,654 (41.52%), 13,525 (41.13%), 21,179 (64.41%) and 13,937 (42.38%) have hits in the KEGG, KOG, Pfam and GO databases, respectively (Table 3). The NR based homology sequences in related species showed that transcript sequences had 36.91%, 24.9%, 2.9%, 2.8% and 1.87% similarity with *Thalassiosira pseudonana, Thalassiosira oceanica,* *Fragilariopsis cylindrus*, *Fistulifera solaris* and *Ricinus communis* (Table 3).

**Figure 1:**
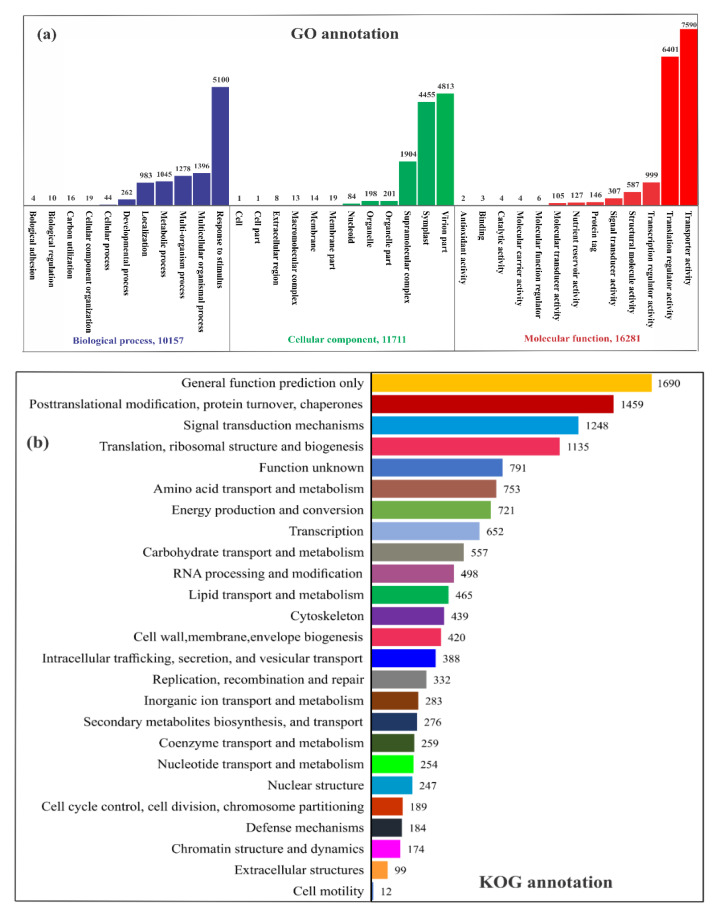
Differential gene expression, and pathway enrichment analysis. (**a**) GO annotation (**b**) KOG annotation

The GO annotation showed 38,149 unigenes categorizations involved with 36 GO classes, in which biological process (11), cellular component (12) and molecular function (13) are the major classes, with 10,157, 11,711 and 16,281 annotated unigenes respectively (Figure 1a). To analyze the unigenes’ intracellular metabolic process, the KOG database was used, indicating that 13,525 unigenes assigned into 25 functional processes (Figure 1b). Among all, amino acid metabolism (753), lipid transport and metabolism (465) and secondary metabolites biosynthesis (276) were the notable functional terms. The distribution of overall fragments mapped (FPKM) for *S. dohrnii* between HC and LC conditions with assembled transcript clusters (unigenes length) is also shown in (Figure S1, and Table S1). The FPKM values were used for the evaluation of the expressed value and quantification of transcripts because it reflects the expression of genes, to which the fragments correspond. We chose a significant threshold of FPKM ≥ 0.1 to prevent analysis with expression levels close to zero for transcripts that had FPKM values < 0.05. Results demonstrated that different unigenes could be supported by different numbers of reads in cells grown at HC and LC conditions. Especially, the ranged FPKM unigenes distribution were from 0.004 to 3.918 in HC conditions, while it was considerably different from 0.004 to 3.718 at LC conditions.

Moreover, we perform unsupervised principal component analysis (PCA) based on the expressed genes, to understand if metabolites differ between these two treatments (Figure S2). The matrix contained 10,974 DETs (active variables) and six samples. The Pearson correlation between high and low carbon triplicate samples ranged from 0.19 to 0.47, shows significant variation between these two conditions. Factor 1 and factor 2 account for 62.83% and 22.79% of the variation, respectively. Factor 1 (cells grown at HC) clearly discriminate metabolic response from cells treated with LC. Both factors consistently cluster together, indicating an influence of different environmental conditions on the cellular development processes.

**Figure 2:**
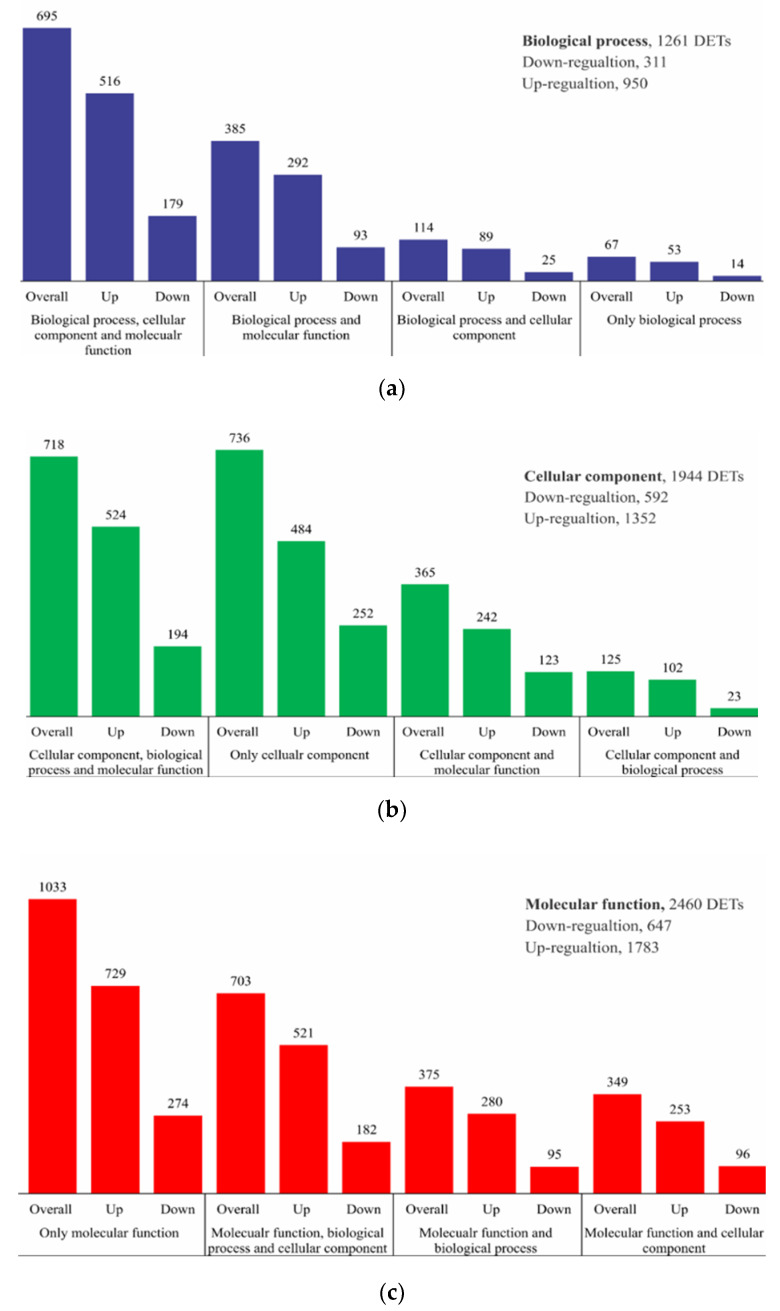
GO enrichment analysis of DETs in *S. dohrnii* between cells treated with HC and LC conditions. Please see Table S2 for the comprehensive GO enrichment analysis of each sample. (**a**) differentially expressed genes in biological process, (**b**) differentially expressed genes in cellular component, (**c**) differentially expressed genes in molecular function.

The combining of temperature and *p*CO_2_ variation in this study exhibits 10,974 differentially expressed transcripts (DETs) comparing between HC and LC treatments. Based on log-2-fold with a false discovery rate (FDR), 0.001, a total of 8414 transcripts were upregulated and 2560 were downregulated in the HC treatment in comparison with the LC treatment (Figure S3). Out of these, all DETs, 5635 unigenes were successfully annotated into GO enrichment analysis to understand the *S. dohrnii* functional response, when treated with HC and LC conditions (Figure 2). The result shows that of the annotated DETs 2460 were involved in the molecular functions, followed by cellular component 1944 and biological process 1261. The membrane transport, lipid and fatty acid biosynthesis were the notably enriched term in the biological process, whereas, cell, membrane and organelle parts are the most enriched term in the cellular process; lipid transport and metabolic process in molecular function were the top enriched terms, respectively.

To further understand the DETs and its involvement in the specific metabolic pathway, we annotated GO enriched genes in KEGG pathway analysis, which represent 3294 transcripts mapped with 127 metabolic pathways (Table S2). The annotated transcripts’ regulation and associated metabolic functions with other databases are shown in Table S2. The DETs showing that metabolism associated ABC transports (*p <* 0.003), chlorophyll metabolism (*p <* 0.001), carotenoid biosynthesis (*p <* 0.001), phenylalanine, tyrosine and tryptophan biosynthesis (*p <* 0.005), phenylpropanoid biosynthesis (*p <* 0.005), lipoic acid (*p <* 0.005), fatty acid biosynthesis (*p <* 0.005) and carbon metabolism (*p <* 0.004) were significant pathways altered by combining temperature and *p*CO_2_ changes in this study. We, therefore, further investigate DETs associated with these metabolisms.

### 2.3. Membrane Transporters

In this study, 53 ABC transporters were differentially expressed consisting of 45 upregulated and 8 downregulated transcripts (Table S3). Of these DETs, membrane transport associated with multidrug/pheromone exporter, lipid exporter (ABC1), peptide exporter, pleiotropic drug resistance proteins, ABC superfamily (breast cancer resistant) and mitoxantrone resistance were the notable upregulated transcripts, whereas, metal transporters and mitochondrial Fe/S exporter were the important downregulated transcripts.

### 2.4. Carotenoid Biosynthesis and Chlorophyll Metabolism

We observed 35 transcripts encoded with carotenoid biosynthesis were differentially expressed in this study, including 24 upregulation and 11 downregulation (Table S4). Based on the KEGG orthologs, violaxanthin de-epoxidase, phytoene desaturase (PDS), beta-ring hydroxylase, beta-carotene isomerase, prolycopene isomerase, zeaxanthin epoxidase (ZEP), and cytochrome P450 (CYPs) enzymes were upregulated by high temperature and *p*CO_2_. In addition, 65 DETs associated with porphyrin and chlorophyll metabolism were also identified in this study, of which 46 transcripts increased and 19 transcripts decreased in abundances (Table S4). Many important enzymes of chlorophyll biosynthesis, i.e., methyltransferases, protoheme Ferro-lyase, porphobilinogen deaminase, glutamyl-tRNA synthetase, and heme oxygenase (HO) were increased in abundances; whereas, isoflavone reductase (IFR), uroporphyrinogen decarboxylase (UROD), and delta-aminolevulinic acid dehydratase (ALAD) were decreased in abundance.

### 2.5. Phenylalanine, Tyrosine and Tryptophan Biosynthesis

Biosynthesis of aromatic amino acids, phenylalanine (Phe), tyrosine (Tyr) and tryptophan (Trp)-associated transcripts were significantly altered in this study by increased temperature and *p*CO_2_ concentration. The DETs encompass these pathways under stress vs. healthy organisms involved in different pathways in a different manner. Results show that a total of 28 transcripts in (Phe) biosynthesis were significantly altered in abundance, of which 24 were upregulated and 4 were downregulated (Table S5). Of these DETs, vital enzymes of kynurenine aminotransferase (KATs), histidinol-phosphate aminotransferase, 3-hydroxyacyl-CoA dehydrogenase, 4-hydroxyphenylpyruvate dioxygenase (HPPD), and Acyl-CoA synthetase were upregulated. Likewise, in tyrosine (Tyr) biosynthesis, 28 DETs, including 24 upregulated and 4 downregulated transcripts, were identified in this study (Table S5). Of these DETs, vital enzymes of aspartate aminotransferase (AST), fumarylacetoacetate hydroxylase (FAH), 4-hydroxy-phenypyruvate dioxygenase, polyphenol oxidase, and homogentisate 1,2-dioxygenase, were upregulated.

Similarly, 42 DETs associated with tryptophan metabolism were identified as differentially expressed, including 37 upregulated and 5 downregulated transcripts (Table S5). Enzymes of aldehyde dehydrogenase (ALDH), glutaryl-CoA dehydrogenase (GCDH), L-kynurenine hydrolase, 2-oxoglutarate dehydrogenase, (OGDC), acetyl-CoA acetyltransferase, glutathione S-transferase (GSTs) and serine protease were upregulated in HC treatments compared with LC condition.

### 2.6. Phenylpropanoid and Flavonoid Biosynthesis

A total of 102 transcripts associated with phenylpropanoid biosynthesis were significantly regulated (Table S6), by elevated temperature and *p*CO_2_, which represent 79 upregulated and 23 downregulated transcripts. The vital upregulated enzymes of these pathways are alcohol dehydrogenase (ADH), pectinesterase (PE), gelatinase, gibberellin-related protein (GAs), collagens, and β-glucosidase. Furthermore, seven transcripts were significantly altered in flavonoid biosynthesis, and all of them were increased in abundance (Table S6), including anthocyanidin synthase (ANS) and caffeoyl-CoA-O-methyltransferase.

### 2.7. Lipid Metabolism and Fatty Acid Biosynthesis

Six transcripts encompassing α-lipoic metabolism were regulated in this study, which consists upregulation of lipoate synthase and lipoyl transferase enzymes (Table S7), whereas seven DETs in ether lipid metabolism have shown upregulation (Table S7), including phospholipase A2 (PLA2s), heterochromatin (HP1) and alpha-beta hydrolase superfamily. Further to this, a total of 90 transcripts associated with fatty acid biosynthesis were significantly regulated in this study, consisting of 74 upregulated and 16 downregulated transcripts (Table S7). The upregulated transcripts of glutaryl-CoA dehydrogenase (GCDH), β-ketoacyl-ACP reductase, long-chain-fatty-acid–CoA ligase, enoyl-CoA hydratase (ECH), acetyl-CoA acetyltransferase, very-long-chain enoyl-CoA reductase, ACP transacylase, fatty acyl-CoA elongase and 3-oxoacyl-acyl-carrier-protein are the important enzymes involved in fatty acid production, while fabA-like domain and AMP-binding enzyme were the notable downregulated enzymes in this metabolism.

## 3. Discussion

Diatoms are promising microorganisms for commercial purposes, as they grow and proliferate rapidly in a short time with highly enriched valuable products naturally [22]. These features make them lead unique candidates as a source of valuable bioproducts, such as pigments, lipids, and bioactive compounds. However, there are still gaps between altering the diatom metabolism for wide-scale commercial applications [23]. Using global transcriptome with modern bioinformatics, here, we investigate the combining effect of increased temperature and *p*CO_2_ concentration on diatom metabolism and their associated biotechnological applications. Despite earlier studies demonstrated elevated temperature and *p*CO_2_ impact on diatoms [6,14], there is no information on *S. dohrnii* for commercial applications, which, if confirmed, could be a significant advancement for wide-scale commercial use of this algae.

Recent reviews demonstrated the influence of elevating temperature and CO_2_ on diatom’s physiology and cellular development [24,25]. Some investigation noted that high temperature and CO_2_ has positive effects on diatom’s physiology [26,27,28] while others noted negative effects [29,30], or unaffected [31,32]. These contradictory findings could be the reason for varied experimental conditions, i.e., different pH [28] light intensities [27] temperature [30] and physiological complexity of diatoms [26,32]. In this study, the combining effect of elevated temperature and *p*CO_2_ stimulated *S. dohrnii* biomass, and pigment contents, which is consistent with other investigations, showed increased growth and biomass of diatoms by elevated temperature and *p*CO_2_ concentration [26,27,32]. The high pigment contents under HC conditions could be the reason for photo-acclimation or a consequence of increasing cell volume rather than an increase in *p*CO_2_ availability [27,31]. Furthermore, consistent with our present study, recent investigation on the diatom *Navicula directa* showed the effect of increasing temperature and *p*CO_2_ promoted the photosynthesis process, pigment composition and growth rate by approximately 43% [30].

Among the DETs, we found significant modulation of ABC transporters, including upregulation of multidrug exporter by 8.08-fold, pleiotropic drug resistance (5.58-fold), breast cancer resistance (9.0-fold) and lipid exporter proteins (7.23-fold). Multidrug proteins are designed by plants to confer resistance to various drugs [33] to protect cells from stress conditions and toxic environments. These multidrug proteins observed earlier in the marine organisms including diatoms, sponges, fish, and mussels, as potential anticancer drugs for human cancer [34,35,36]; therefore, over expression of these transporters in this study by combining temperature and *p*CO_2_ elevation could be a valuable source for the biotechnological application.

Pigments composition is highly sensitive to environmental stress, including temperature [14] pH [16], and even combining temperature and *p*CO_2_ changes [30]. Consistently, upregulated phytoene desaturase (10.36-fold) in this study by elevated temperature and *p*CO_2,_ increased ketocarotenoids accumulation during changes of temperature and pH together. A similar finding was seen earlier in *Synechococcus sp* [36] and *Haematococous sp* [37] response to higher temperature. Further to this, it was reported that pH variation increased the violaxanthin de-epoxidase enzyme to accumulate zeaxanthin on model diatom *P. tricornutum* [38]. Likewise, pH variation in this study increased the abundance of violaxanthin de-epoxidase (2.76-fold), and elevated zeaxanthin epoxidase by (3.71-fold), which can be extracted for industrial application. Beta-carotene upregulation (2.31-fold) in this study plays an important role by protecting cells from free radicals involved in light-harvesting functions [39]. However, the earlier investigation noted β-carotene as a potential antitumor agent in humans [40]. In agreement with these results, Nishino et al. [41] reported that carotenoid contents, including β-carotene, zeaxanthin and violaxanthin de-epoxidase exhibit greater anti-carcinogenic activity in the market. Besides, the upregulation of methyltransferase (2.05-fold) in chlorophyll metabolism would also promote betaine lipid biosynthesis and those were earlier found to be upregulated in a diatom under phosphate stress [42]. While other upregulated transcripts in chlorophyll metabolism by combining temperature and *p*CO_2_ variation in this study are involved in heme-binding larger chloroplast, those were earlier found to be regulated under phosphate [43] and nitrate deprivation on diatoms [44].

In green algae and higher plants, three aromatic amino acids (Phe), (Tyr) and (Try) are derived from the common precursors of the Shikimate pathway; those were similar in diatoms based on the gene homology [45]. These amino acids are known to be essential products for human food and pharmacological industries [46]. In diatoms, amino acid biosynthesis metabolism could be strictly regulating by stress conditions [45]; as a consequence, the commitment enzymes to relieve or eliminate the end products are crucial to increasing target compounds [45]. In this study, wide regulation of enzymes associated with phenylalanine (Phe) metabolism was upregulated, suggesting increased (Phe) end products for commercial purposes from *S. dohrnii.* A similar investigation was carried out earlier on eukaryotes by stress conditions, resulting in increased end product of (Phe) [47]. A previous study on *P. tricornutum* upon nitrate assimilation [15] revealed an upregulated aspartate aminotransferase enzyme that remodels the amino acid metabolism and its concentration. Consistently, the upregulation of this aspartate aminotransferase (7.76-fold) in this study would be expected to do the same functions under combining temperature and CO_2_ elevation, whereas other upregulated transcripts, are important for the precursor of variant amino acids and derived metabolites for the cellular growth, and defense mechanism during stress conditions [48]. Moreover, it was reported that increased concentrations of these three aromatic amino acids resulting in elevation of plant secondary metabolites, could be used as valuable products [49,50]. Similarly, we predict the same results by triggering these three amino acid biosynthesis alterations in *S. dohrnii* by elevated temperature and *p*CO_2_ concentration.

Our results showed that elevation of temperature along with *p*CO_2_ influenced on amino acids composition. This finding agrees with previous results on the diatom *Cylindrotheca fusiformis* [51], green algae *Chlorella* strain MFD-1 and *Nannochloropsis* strain MFD-2, which showed increasing amino acids accumulation up to 36% and 38% respectively, when temperature alone shifted from 15 to 25 °C [52]. The results of these studies indicate that temperature elevation alone can increase AA composition in algae because temperature has greater influence on algal physiological process than CO_2_ variation [51,52]. This could be due to temperature involvement in a high number of metabolic process, i.e., enzymatic reactions and photosynthesis process, that subsequently lead to high growth and metabolic rates [53] of the cell than CO_2_. Consequently, the amount of synthesized protein and AA composition by temperature influenced metabolic pathways higher than what is produced by CO_2_ influenced mechanisms [51]. Nevertheless, these findings do not describe why proportionally, AA associated genes are more increased in this study by both elevating temperature and CO_2_ changes. A detailed investigation exclusively on AA metabolism of these individual stress condition will be required to clarify these effects.

Recent investigations on *S. marinoi* revealed upregulation of ALDH enzyme with associate production of ethanol, drug biotransformation and oxylipin production [4]. Consistently, this enzyme was upregulated (5.48-fold) in this study, by combining elevated temperature and *p*CO_2_, suggesting possible enhancement of these above-mentioned by-products from *S. dohrnii.* Yamauchi et al. found that the upregulation of (GAs) in *A. thaliana* response to a lower temperature was involved in higher ethylene production [50], which is widely used for the chemical industry. Consistently, varied temperatures in this study increased this gibberellin-regulated protein (1.46-fold), suggesting possible development of ethylene production. Likewise, other upregulated transcripts in phenylpropanoid metabolism, including beta-glucosidase (4.99-fold), pectinesterase (4.87-fold), and gelatinase (2.07-fold) would promote the efficiency of glucose production, cell wall modification, and other amino acids compounds of *S. dohrnii.*

In recent years, the evaluation of genomic approaches targeted crop plants for the high efficiency of flavonoid biosynthesis and associated valuable products. These genetically modified flavonoid pathways in higher plants can be achieved as follows: (i) upregulating transcripts to increase the carbon flow direction towards the target products and (ii) upregulating transcripts involved in competing pathways and identifying exogenous genes towards the biosynthesis of new products [54]. Such edited transcripts evidenced increasing health-promoting pigments [55] and pharmacological products in higher plants [56]. Similarly, upregulating flavonoid biosynthesis transcripts in this study by stress condition expected to favor commercial purposes.

The effect of temperature and CO_2_ variation on lipid content and fatty acid composition of diatoms [6] and other microalgae have been previously reported [57] and showed increased accumulation of these contents under these stress conditions. Accordingly, in this study, the total lipid content and productivity increased in cells grown under combining high temperature and CO_2_. Some studies showed that the ratio of these accumulated bioproducts significantly enhanced by the stress conditions, i.e., nutrients [58], temperature [14] and pH variation [16] to direct carbon sources for commercial products. Indeed, being photoautotrophic to accumulate the lipid and fatty acids during stress, diatoms required carbon sources. Because diatoms use polysaccharide, chrysolaminaran (β-1,3-gluvan) during normal conditions as their carbon sink [59], while during stress, the carbon flux reprogrammed towards lipid synthesis and stored as TAGs [60]. Consistently in this study, many upregulated acyl-CoAs by temperature and CO_2_ variation could be used for TAG synthesis, resulting in increased lipid content under high temperature and CO_2_ conditions. These findings suggest that under these combining temperature and CO_2_ conditions_,_ *S. dohrnii*’s main direction of carbon flow is towards the lipid and fatty acid accumulation. Such energy exchange of different diatoms was previously tested by Wu et al. [61] under elevated *p*CO_2_ concentrations. This study observed that diatoms that grew with high CO_2_ levels decreased carbon metabolism to save energy to increase growth rates and lipid productivity. This finding suggests that elevated CO_2_ in this study can also induce the carbon flux towards lipid biosynthesis, which could be a useful source for wide-scale industrial applications.

The fatty acid profile diatoms are highly rich in monounsaturated acids (MUFAs) to produce biodiesel; nevertheless, properties of biodiesel are determined by carbon chain length and unsaturation extent presence in the fuel [6,13]. This tendency was also identified in this study by upregulating many long-chain fatty acids proteins (which have 20 or more carbon atoms) and downregulating saturating enzymes in fatty acid metabolism. This increasing unsaturated fatty acid and decreasing saturated fatty acids have been observed recently on *T. pseudonana*, with respect to elevated CO_2_ for biodiesel production [6]. However, this trend was not identified in other species [62], showing a unique feature of *S. dohrnii* for biotechnological application under the combining of elevated temperature and CO_2_ concentration. Similarly, the combining effects of changing temperature and CO_2_ were also reported on the diatom *Nitzschia lecointei*, where cellular PUFA content regulated up to 40% [30].

Despite the unclear mechanisms of elevated CO_2_ influence on diatom FA metabolism, it was predicted that regulated saturated fatty acids (SFA) in cells grown at high CO_2_ environment is due to FA synthesis and accumulation [63]. It has been noted that high CO_2_ levels with low pH in algae can severely regulate the cellular pH, thereby distributing tightly regulated homeostasis [64]. Subsequently, regulating SFA synthesis under high CO_2_ environment can be a less fluid cell membrane produce mechanism, which is built of short-chained FA, making them less permeable and helping in variation of cellular homeostasis [65]. Besides, temperature can also affect the FA profile of algae [30,66]. Our investigation showed significant temperature related effect on FA metabolic genes of *S. dohrnii* with CO_2_ changes, with a higher amount of upregulated transcripts in HC conditions compared with LC conditions. These results agree with the FA profile of the diatom *Chaetoceros muelleri*, which reveled variance of PUFA content when temperature changed 5 °C at culture condition [66]. Nevertheless, our results are contradictory to previous investigations, in which PUFA concentrations are commonly inversely proportional to temperature variation [67]. This may be the reason of the length of experiment and allocation of carbon sources within the cells as mentioned above.

In addition, recent years of genetic engineering on diatoms demonstrated increasing lipid content and fatty acid composition using overexpression, and silencing transcripts modification [68]. Especially, stimulating fatty acid-elongase, D5-desaturase, Acyl-ACP, and Acetyl-CoA carboxylase were demonstrated to increase the lipid and fatty acids contents [69,70], while transcripts associated with multi-functional lipase/acyltransferase, pyruvate metabolism were silenced to the increased yield of lipid contents [71,72]. Remarkably, all these transcripts were up and downregulated respectively in this study by combining elevated temperature and *p*CO_2_, promising the significant potential of *S. dohrnii* for the biodiesel production without genetic modification. We show here, *S. dohrnii* grown well with biomass, bioactive compounds, and lipid productivity under combining elevated temperature and *p*CO_2_ condition, showed the unique features of CO_2_ biofixation capability; collectively, suggesting the significant potential of this species to be cultivated in both in the laboratory and open bonds using elevating temperature and CO_2_ for wide-scale biotechnological applications.

## 4. Materials and Methods

### 4.1. Experimental Setup, Species Collection and Culture Condition

To reduce contamination, accurate methods were used in this study. Briefly, a culture experiment was conducted in 5 L polycarbonate bottles (3 + 3 = 6 nos), which had been soaked for 1 week in 1% Citranox^®^ and 2 weeks in 1 mol L^−^^1^ hydrochloric acid (HPLC) grade. The bottles were rinsed seven times with ultra-pure water (Merck Millipore Corporation) between each soaking step. Finally, the cleaned bottles were air-dried under a clean desk and packed with three polyethylene bags for storage prior to beginning the experiment. This investigation was conducted with globally abundant, and ecologically important bloom-forming marine diatom *Skeletonema dohrnii*, which was isolated from the Yellow sea [20]. The isolated strain was maintained at Artificial Sea Water medium according to the Aquil recipe [73] at 21 °C with (~400 ppm) 12 h:12 h light: dark cycle, with a light intensity of 100 μmol m^−2^ s^−1^ using cool white fluorescent tubes [20]. For the experiment, two different levels of temperature (21 and 25 °C) and pCO_2_ culture conditions (400 and 1000 ppm) were set, referred to as LC (400 ppm, 21 °C normal) and HC (1000 ppm, 25 °C stress condition) respectively, were grown in the above-mentioned conditions, and were gently bubbled pCO_2_ through filtration (0.22 µm) using CO_2_ incubators [26]. The LC and HC conditions represent the present and future (2100 years) atmospheric CO_2_ concentrations [74]. The duration of the overall experiment was eight days; nevertheless, in each treatment (LC and HC), triplicate samples were collected during the mid-exponential stage (day 4) for all the physiological analyses.

### 4.2. Seawater Carbonate System and Parameters

During experiment to ensure stable seawater carbonate chemistry (pH variation < 0.05), culture was diluted every 24 h using pre-CO_2_ equilibrated medium and the cell density was maintained at no more than 10 µg chlorophyll-a L^−1^. The pH variation of culture was monitored every day prior to dilution using pH probe (Mettler Toledo DL 15 Titrator, Stockholm, Sweden), calibrated with National Bureau of Standard (NBS) buffer solutions of pH 7.0 and 10 (Sigma-Aldrich, St Louis, MO, USA). DIC was measured using Shimadzu Total Organic Carbon Analyzer (TOC-5000A, Kyoto, Japan) and total alkalinity (TA) was measured by potentiometric titration. The other parameters of the carbonate system were calculated using CO_2_SYS software based on the known values of DIC, pH, salinity, alkalinity, and temperature [75].

### 4.3. Determination of Growth Rate and Cell Density

The initial cell density was 5 × 10^5^ (cells mL^−1^) at the beginning of experiment. In this study, cell density was determined daily using a Beckman Multisizer ™ 3 Coulter Counter^®^ with a 100 μm aperture. Growth rate *µ*(d^−1^) was calculated as follows:

*µ* = 1n (*N*_t_:*N*_0_)/*t* (1)

where, *N*_0_ and *N*_t_ are at the end and start of the exponential phase of growth, respectively, and *t* is the duration of the exponential growth phase.

### 4.4. Determination of Pigments

Fifty mL of culture samples, grown under LC and HC conditions, were filtered onto GF/F membrane (25mm, Millipore, MA, USA) and placed overnight in a refrigerator (4 °C) after adding 10 mL of methanol. Consequently, the samples were centrifuged (5000× *g*, 10 min), and the supernatant was measured using a microplate reader (Thermo, Multiskan, GO, USA). The final concentrations of carotenoid and chlorophyll were calculated following Gao et al. [76].

### 4.5. Determination of Biochemical Analysis

The protein content of *S. dohrnii* was determined by the Bradford method [77] and the total carbohydrate was measured by the Anthrone-Sulphuric Acid Colorimetric method [78]. Further to this, a modified Folch method [79] was conducted to determine the lipid contents of cells as follows. Approximately, 4 L of algal cells at each sample was harvested by centrifugation (7500× *g*, 5 min) and dried in an oven at 60 °C to a constant weight. Fifty (mg) of grinded algae powder was transferred to a 10 mL centrifuge tube, with added solutions of 5 mL chloroform-methanol mixture and (V:V = 2:1) and 1 mL NaCl (0.88%) respectively. After being shaken by a multi-tube vortex mixer (DMT-2500, Zhengzhou, China) for 20 min (2000 rpm), the solution was centrifuged (5000× *g*, 5 min) and the supernatant was removed; subsequently, 5 mL of methanol-water solution (V:V) was added to at 25 °C trifuge tube. After centrifuging and removing the supernatant one more time, the bottom phase was dried using a steady stream of nitrogen supplied by the nitrogen blowing device (Autoscience, MTN-5800, Tianjin, China). Lipid content (pg cell^−1^) was calculated based on the lipid weight and cell number/weight. Lipid productivity (mg L^−1^) was determined by the changes in lipid production for two days of comparison periods.

### 4.6. RNA Isolation

The complete experimental and carried out bioinformatics analysis of this study are shown in (Figure S4). Initially, 1 L of cultures grown with high and low *p*CO_2_ cells was harvested (3 + 3 = 6 L) by gentle filtration onto 0.8 μm polycarbonate filters (Millipore, MA, USA) during the mid-exponential stage. Filtered cells were immediately flash-frozen with liquid nitrogen and stored at −80 °C until RNA extraction. Total RNA was extracted from frozen cells using Trizol reagents (Invitrogen, Waltham, MA, USA) following the manufacturer’s protocol. The purity and concertation of RNA were checked using the NanoDrop-6000 Assay-Kit and an Agilent 2100 Bioanalyzer system (Agilent Technologies, Santa Clara, CA, USA). An approximation of 5 µg of RNA used as input materials for cDNA library construction. The overall unigenes’ FPKM distribution and density variation among six samples are given in Figure S1.

### 4.7. Library Construction and Sequencing

As there is no genome DNA available of these species, the construction and sequencing of the cDNA library in this study were performed as follows. Briefly, the poly (A) messenger RNA was isolated from total RNA samples with oligo (dT) attached magnetic beads (Illumina, San Diego, CA, USA) and using divalent cation fragmentation buffer incubated at 94 °C for five minutes, where the mRNA was fragmented into short fragments. The cleaved RNA fragments were then reverse transcribed to the first strand cDNAs by random hexamer primers. Following this, the second strand cDNAs were synthesized to construct the final cDNA library using the Illumina platform (San Diego, CA, USA). After the final repairing process and ligation of the adapter, RNA was amplified by using PCR and purified by QIAquick Gel Extraction Kit (Qiagen, Venlo, Netherlands). The cDNA library of *S. dohrnii* tissue was sequenced on the Illumina Hiseq 2000 platform (San Diego, CA, USA) with paired-end reads of 90 nucleotides.

### 4.8. Quality Control and de Novo Assembly

Prior to de novo assembly, we removed low-quality raw reads, and PCR duplicates using PrintSeq v0.20.4 (San Diego, CA, USA); adapters were clipped using Trimmomatics v0.38 in [80]. De novo assembly was performed using Trinity v2.8.4 [81], based on the obtained high-quality clean reads. Further to this, using TransDecoder v5.5.0 (https://transdeooder.github.io/), the raw assembly results were filtered for a minimum transcript length of 300 nucleotides and detectable CDS. The CDS of all the similar alternative splice-form (transcripts) sets was selected as a unigenes; the completeness of all the assembly was assessed using BUSCO v3 [82].

### 4.9. Functional Annotation, DETs Detection and Pathway Enrichment Analysis

The resulting transcripts were annotated using InterProscan v5.33-72.0 [83], to NCBI-NT and NCBI-NR [84], Pfam databases [85] Swiss-Prot, KOG, GO, and KEGG [86]. Furthermore, with a (FDR < 0.01 and fold change > 2), the differentially expressed transcripts (DETs) in this study were obtained based on the methods of empirical Bayesian (fold change > 2 and posterior probability of being equivalent expression < 0.05) [87], noisy distribution (fold change > 2 and probability > 0.8) [88], negative binomial distribution [89], and Poisson distribution (fold change >2 and FDR < 0.001) [90]. The metabolic pathway analysis of differentially expressed transcripts was conducted according to the KEGG pathway database (http://www.genome.jp/kegg/) and the functional enrichment analysis was performed using phyper, a function of R software. The *p*-value calculating formula in the hypergeometric test is:

(2) $$P=1-\overset{m-1}{\underset{i=0}{\sum }}-\frac{\left(\begin{array}{c} M\\ i \end{array}\right)\left(\begin{array}{c} N\\ n \end{array}\begin{array}{c} -\\ - \end{array}\begin{array}{c} M\\ i \end{array}\right)}{\left(\begin{array}{c} N\\ n \end{array}\right)}$$

Then, we calculate the false discovery rate (FDR) for each *p*-value; in general, the terms where FDR is no larger than 0.01 are defined as significantly enriched.

### 4.10. Statistical Analysis

Statistical analysis of all the physiological data was performed with unpaired *t*-test analysis using GraphPad Prism 6 software. Triplicate samples of each condition were taken into consideration, and comparison was done between groups of cells grown at high temperature and *p*CO_2_ versus low temperature and *p*CO_2_.

## 5. Conclusions

In this study, utilizing combined elevated temperature and *p*CO_2,_ the whole-cell transcriptome profile of *S. dohrnii* was investigated for the first time to evaluate the metabolic potential associated with biotechnological applications. We showed under elevated temperature and *p*CO_2_ conditions, *S. dohrnii* grew well with higher biomass, bioactive components synthesis and lipid productivity using the unique CO_2_ biofixation ability. Our study also provides evidence of a new potential organism for their bioactive compound and natural products, offering a new exploration of promising algae to microalgal-based derived bioactive compounds for wide-scale biotechnological applications.

## Figures and Tables

**Table 1:** Physiological parameters of diatom *S. dohrnii* grown at LC and HC environmental conditions LC (21 °C, 400 ppm) and HC (25 °C, 1000 ppm).

	LC (21 °C 400 ppm)	HC (25 °C 1000 ppm)
Growth rate (day^−1^)	0.76 ± 0.05 *	1.23 ± 0.15
Cell density (10^4^ cells mL^−1^)	233.3 ± 9.07 *	297.3 ± 9.71
Chlorophyll *a* (pg cell^−1^)	0.20 ± 0.01 *	0.28 ± 0.02
Carotenoid (pg cell^−1^)	0.05 ± 0.07 *	0.08 ± 0.09
Protein content (pg cell^−1^)	3.1 ± 0.2 *	3.7 ± 0.1
Carbohydrate (pg cell^−1^)	0.07 ± 0.5	1.4 ± 0.9
Lipid content (pg cell^−1^)	13.33 ± 1.5 *	19.27 ± 1.5
Lipid productivity (mg L^−1^)	12.51 ± 0.5 *	17.35 ± 0.6

All data are shown as average (*n* = 3) ± S.D. The initial cell density was 5 × 10^5^ (cells mL^−^^1^). at the beginning of experiment. Significant differences (*p* < 0.05), between the treatments are indicated by an asterisk of diatom at exponential stage of given species determined utilizing, *t*-test. * significant differences between the treatment.

**Table 2:** Seawater carbonate chemistry. DIC: dissolved inorganic carbon; HCO_3_^−^: bicarbonate; TA: total alkalinity. All data are shown as average (*n* = 3) ± S.D.

	pH_NBS_	DIC (µmol Kg^−1^)	HCO_3_^−^ (µmol Kg^−1^)	CO_3_^−2^ (µmol Kg^−1^)	CO_2_ (µmol Kg^−1^)	TA (µmol Kg^−1^)
LC (21 °C 400 ppm)	8.12 ± 0.02	2103 ± 11	1894 ± 14	219 ± 6.0	13.6 ± 0.2	2355 ± 16
HC (25 °C 1000 ppm)	7.82 ± 0.01	2248 ± 17	2216 ± 22	126 ± 3.1	31.4 ± 1.0	2347 ± 14

**Table 3:** Functional annotation of the identified unigenes.

Functional Annotation			NR Homology	
	Number	Percentage	Species	Percentage
Total	32,884	100	*Thalassiosira pseudonana CCMP 1335*	36.91
NR	22,261	67.70	*Thalassiosira oceanica*	24.90
NT	2960	9.00	*Fragilariopsis cylindrus CCMP 1102*	2.90
Swiss-Prot	11,573	35.19	*Fistulifera solaris*	2.80
KEGG	13,654	41.52	*Ricinus communis*	1.87
KOG	13,525	41.13	Others	30.62
Pfam	21,179	64.41		
GO	13,937	42.38		
Intersection	1487	4.52		
Overall	25,332	77.03		

## Supplementary Materials

The following are available online at https://www.mdpi.com/1660-3397/18/5/259/s1, Table S1: de novo assembles, and RPKM distribution details, Table S2: GO enrichment and KEGG pathway enrichment analysis results, Table S3: Differentially expressed ABC transporters, Table S4: Differentially expressed transcripts in carotenoid biosynthesis and chlorophyll metabolism, Table S5: Differentially expressed transcripts in phenylalanine, tyrosine and tryptophan biosynthesis, Table S6: Differentially expressed transcripts in phenylpropanoid and flavonoid biosynthesis, Table S7: Differentially expressed transcripts in lipid metabolism and fatty acid biosynthesis. Supplementary Figures: FPKM distribution (S1), PCA analysis (S2), DETs profiling (S3); experimental setup and carried out bioinformatics pipeline (S4).

## References

[1] Batista A.P., Gouveia L., Bandarra N.M., Franco J.M., Raymundo A. (2013). Comparison of microalgal biomass profiles as novel functional ingredient for food products. Algal Res..

[2] Lum K.K., Kim J., Lei X.G. (2013). Dual potential of microalgae as a sustainable biofuel feedstock and animal feed. J. Anim. Sci. Biotechnol..

[3] Wang K., Wommack K.E., Chen F. (2011). Abundance and distribution of Synechococcus spp. and cyanophages in the Chesapeake Bay. Appl. Environ. Microbiol..

[4] Lauritano C., Carotenuto Y., Vitiello V., Buttino I., Romano G., Hwang J.S., Ianora A. (2015). Effects of the oxylipin-producing diatom Skeletonema marinoi on gene expression levels of the calanoid copepod Calanus sinicus. Mar. Genom..

[5] Hamed I., Özogul F., Özogul Y., Regenstein J.M. (2015). Marine Bioactive Compounds and Their Health Benefits: A Review. Compr. Rev. Food Sci. Food Saf..

[6] Sabia A., Clavero E., Pancaldi S., Salvado Rovira J. (2018). Effect of different CO2 concentrations on biomass, pigment content, and lipid production of the marine diatom Thalassiosira pseudonana. Appl. Microbiol. Biotechnol..

[7] Ekinci K., Erdal I., Uysal Ö., Uysal F.Ö., Tunce H., Doğan A. (2018). Anaerobic Digestion of Three Microalgae Biomasses and Assessment of Digestates as Biofertilizer for Plant Growth. Environ. Prog. Sustain. Energy.

[8] Rashid N., Park W.K., Selvaratnam T. (2018). Binary culture of microalgae as an integrated approach for enhanced biomass and metabolites productivity, wastewater treatment, and bioflocculation. Chemosphere.

[9] Mondal M., Goswami S., Ghosh A., Oinam G., Tiwari O., Das P., Gayen K., Mandal M., Halder G. (2017). Production of biodiesel from microalgae through biological carbon capture: A review. 3 Biotech.

[10] Trobajo R., Ibañez C., Clavero E., Salvadó J., Jørgensen S.E. (2014). Modelling the response of microalgae to CO2 addition. Ecol. Model..

[11] Nascimento I.A., Cabanelas I.T.D., Santos J.N., Nascimento M.A., Sousa L., Sansone G. (2015). Biodiesel yields and fuel quality as criteria for algal-feedstock selection: Effects of CO2-supplementation and nutrient levels in cultures. Algal Res..

[12] Hildebrand M., Davis A.K., Smith S.R., Traller J.C., Abbriano R. (2012). The place of diatoms in the biofuels industry. Biofuels.

[13] Joseph M.M., Renjith K., John G., Nair S.M., Chandramohanakumar N. (2017). Biodiesel prospective of five diatom strains using growth parameters and fatty acid profiles. Biofuels.

[14] Kurpan Nogueira D.P., Silva A.F., Araújo O.Q.F., Chaloub R.M. (2015). Impact of temperature and light intensity on triacylglycerol accumulation in marine microalgae. Biomass Bioenergy.

[15] Smith S.R., Dupont C.L., McCarthy J.K., Broddrick J.T., Oborník M., Horák A., Füssy Z., Cihlář J., Kleessen S., Zheng H. (2019). Evolution and regulation of nitrogen flux through compartmentalized metabolic networks in a marine diatom. Nat. Commun..

[16] Mus F., Toussaint J.P., Cooksey K.E., Fields M.W., Gerlach R., Peyton B.M., Carlson R.P. (2013). Physiological and molecular analysis of carbon source supplementation and pH stress-induced lipid accumulation in the marine diatom Phaeodactylum tricornutum. Appl. Microbiol. Biotechnol..

[17] Di Dato V., Di Costanzo F., Barbarinaldi R., Perna A., Ianora A., Romano G. (2019). Unveiling the presence of biosynthetic pathways for bioactive compounds in the Thalassiosira rotula transcriptome. Sci. Rep..

[18] Muhseen Z.T., Xiong Q., Chen Z., Ge F. (2015). Proteomics studies on stress responses in diatoms. Proteomics.

[19] Heydarizadeh P., Boureba W., Zahedi M., Huang B., Moreau B., Lukomska E., Couzinet-Mossion A., Wielgosz-Collin G., Martin-Jézéquel V., Bougaran G. (2017). Response of CO2-starved diatom Phaeodactylum tricornutum to light intensity transition. Philos. Trans. R. Soc. B Biol. Sci..

[20] Thangaraj S., Shang X., Sun J., Liu H. (2019). Quantitative Proteomic Analysis Reveals Novel Insights into Intracellular Silicate Stress-Responsive Mechanisms in the Diatom Skeletonema dohrnii. Int. J. Mol. Sci..

[21] Yao G., Peng C., Zhu Y., Fan C., Jiang H., Chen J., Cao Y., Shi Q. (2019). High-Throughput Identification and Analysis of Novel Conotoxins from Three Vermivorous Cone Snails by Transcriptome Sequencing. Mar. Drugs.

[22] Wang J.K., Seibert M. (2017). Prospects for commercial production of diatoms. Biotechnol. Biofuels.

[23] Fu W., Wichuk K., Brynjolfsson S. (2015). Developing diatoms for value-added products: Challenges and opportunities. New Biotechnol..

[24] Gao K., Campbell D.A. (2014). Photophysiological responses of marine diatoms to elevated CO2 and decreased pH: A review. Funct. Plant Biol..

[25] Gao K., Beardall J., Häder D.-P., Hall-Spencer J.M., Gao G., Hutchins D.A. (2019). Effects of Ocean Acidification on Marine Photosynthetic Organisms Under the Concurrent Influences of Warming, UV Radiation, and Deoxygenation. Front. Mar. Sci..

[26] Sun J., Hutchins D.A., Feng Y., Seubert E.L., Caron D.A., Fu F.-X. (2011). Effects of changingpCO2and phosphate availability on domoic acid production and physiology of the marine harmful bloom diatomPseudo-nitzschia multiseries. Limnol. Oceanogr..

[27] Li G., Campbell D.A. (2013). Rising CO2 interacts with growth light and growth rate to alter photosystem II photoinactivation of the coastal diatom Thalassiosira pseudonana. PLoS ONE.

[28] Wu Y., Gao K., Riebesell U. (2010). CO2-induced seawater acidification affects physiological performance of the marine diatom Phaeodactylum tricornutum. Biogeosciences.

[29] Mejía L.M., Isensee K., Méndez-Vicente A., Pisonero J., Shimizu N., González C., Monteleone B., Stoll H. (2013). B content and Si/C ratios from cultured diatoms (Thalassiosira pseudonana and Thalassiosira weissflogii): Relationship to seawater pH and diatom carbon acquisition. Geochim. Cosmochim. Acta.

[30] Torstensson A., Chierici M., Wulff A. (2011). The influence of increased temperature and carbon dioxide levels on the benthic/sea ice diatom Navicula directa. Polar Biol..

[31] Crawfurd K.J., Raven J.A., Wheeler G.L., Baxter E.J., Joint I. (2011). The response of Thalassiosira pseudonana to long-term exposure to increased CO2 and decreased pH. PLoS ONE.

[32] Yang G., Gao K. (2012). Physiological responses of the marine diatom Thalassiosira pseudonana to increased pCO2 and seawater acidity. Mar. Environ. Res..

[33] Jasinski M., Ducos E., Martinoia E., Boutry M. (2003). The ATP-binding cassette transporters: Structure, function, and gene family comparison between rice and Arabidopsis. Plant Physiol..

[34] Kurelec B. (1992). The multixenobiotic resistance mechanism in aquatic organisms. Crit. Rev. Toxicol..

[35] Scherer C., Wiltshire K., Bickmeyer U. (2008). Inhibition of multidrug resistance transporters in the diatom Thalassiosira rotula facilitates dye staining. Plant Physiol. Biochem..

[36] Krishna R., Mayer L.D. (2000). Multidrug resistance (MDR) in cancer: Mechanisms, reversal using modulators of MDR and the role of MDR modulators in influencing the pharmacokinetics of anticancer drugs. Eur. J. Pharm. Sci..

[37] Schafer L., Sandmann M., Woitsch S., Sandmann G. (2006). Coordinate up-regulation of carotenoid biosynthesis as a response to light stress in Synechococcus PCC7942. Plant Cell Environ..

[38] Grünewald K., Eckert M., Hirschberg J., Hagen C. (2000). Phytoene desaturase is localized exclusively in the chloroplast and up-regulated at the mRNA level during accumulation of secondary carotenoids in Haematococcus pluvialis (Volvocales, Chlorophyceae). Plant Physiol..

[39] Jakob T., Goss R., Wilhelm C. (2001). Unusual pH-dependence of diadinoxanthin de-epoxidase activation causes chlororespiratory induced accumulation of diatoxanthin in the diatom Phaeodactylum tricornutum. J. Plant Physiol..

[40] Takaichi S. (2011). Carotenoids in algae: Distributions, biosyntheses and functions. Mar. Drugs.

[41] Nishino H., Murakoshi M., Ii T., Takemura M., Kuchide M., Kanazawa M., Mou X.Y., Wada S., Masuda M., Ohsaka Y. (2002). Carotenoids in cancer chemoprevention. Cancer Metastasis Rev..

[42] Dyhrman S.T., Jenkins B.D., Rynearson T.A., Saito M.A., Mercier M.L., Alexander H., Whitney L.P., Drzewianowski A., Bulygin V.V., Bertrand E.M. (2012). The transcriptome and proteome of the diatom Thalassiosira pseudonana reveal a diverse phosphorus stress response. PLoS ONE.

[43] Chen X.H., Li Y.Y., Zhang H., Liu J.L., Xie Z.X., Lin L., Wang D.Z. (2018). Quantitative Proteomics Reveals Common and Specific Responses of a Marine Diatom Thalassiosira pseudonana to Different Macronutrient Deficiencies. Front. Microbiol..

[44] Alipanah L., Rohloff J., Winge P., Bones A.M., Brembu T. (2015). Whole-cell response to nitrogen deprivation in the diatom Phaeodactylum tricornutum. J. Exp. Bot..

[45] Bromke M.A. (2013). Amino Acid biosynthesis pathways in diatoms. Metabolites.

[46] Zhou L., Li F., Xu H.-B., Luo C.-X., Wu H.-Y., Zhu M.-M., Lu W., Ji X., Zhou Q.-G., Zhu D.-Y. (2010). Treatment of cerebral ischemia by disrupting ischemia-induced interaction of nNOS with PSD-95. Nat. Med..

[47] Yin W.B., Amaike S., Wohlbach D.J., Gasch A.P., Chiang Y.M., Wang C.C., Bok J.W., Rohlfs M., Keller N.P. (2012). An Aspergillus nidulans bZIP response pathway hardwired for defensive secondary metabolism operates through aflR. Mol. Microbiol..

[48] De la Torre F., Canas R.A., Pascual M.B., Avila C., Canovas F.M. (2014). Plastidic aspartate aminotransferases and the biosynthesis of essential amino acids in plants. J. Exp. Bot..

[49] Pyne M.E., Narcross L., Martin V.J.J. (2019). Engineering Plant Secondary Metabolism in Microbial Systems. Plant Physiol..

[50] Yamauchi Y., Ogawa M., Kuwahara A., Hanada A., Kamiya Y., Yamaguchi S. (2004). Activation of gibberellin biosynthesis and response pathways by low temperature during imbibition of Arabidopsis thaliana seeds. Plant Cell.

[51] Bermudez R., Feng Y., Roleda M.Y., Tatters A.O., Hutchins D.A., Larsen T., Boyd P.W., Hurd C.L., Riebesell U., Winder M. (2015). Long-Term Conditioning to Elevated pCO2 and Warming Influences the Fatty and Amino Acid Composition of the Diatom Cylindrotheca fusiformis. PLoS ONE.

[52] James C., Al-Hinty S., Salman A. (1989). Growth and ω3 fatty acid and amino acid composition of microalgae under different temperature regimes. Aquaculture.

[53] Taucher J., Jones J., James A., Brzezinski M., Carlson C., Riebesell U., Passow U. (2015). Combined effects of CO2 and temperature on carbon uptake and partitioning by the marine diatoms T halassiosira weissflogii and D actyliosolen fragilissimus. Limnol. Oceanogr..

[54] Mouradov A., Spangenberg G. (2014). Flavonoids: A metabolic network mediating plants adaptation to their real estate. Front. Plant Sci..

[55] Butelli E., Titta L., Giorgio M., Mock H.-P., Matros A., Peterek S., Schijlen E.G., Hall R.D., Bovy A.G., Luo J. (2008). Enrichment of tomato fruit with health-promoting anthocyanins by expression of select transcription factors. Nat. Biotechnol..

[56] Jiang Y., Hu Y., Wang B., Wu T. (2014). Bivalent RNA interference to increase isoflavone biosynthesis in soybean (Glycine max). Braz. Arch. Biol. Technol..

[57] Wang X.W., Liang J.R., Luo C.S., Chen C.P., Gao Y.H. (2014). Biomass, total lipid production, and fatty acid composition of the marine diatom Chaetoceros muelleri in response to different CO2 levels. Bioresour. Technol..

[58] Abida H., Dolch L.J., Mei C., Villanova V., Conte M., Block M.A., Finazzi G., Bastien O., Tirichine L., Bowler C. (2015). Membrane glycerolipid remodeling triggered by nitrogen and phosphorus starvation in Phaeodactylum tricornutum. Plant Physiol..

[59] Kroth P.G., Chiovitti A., Gruber A., Martin-Jezequel V., Mock T., Parker M.S., Stanley M.S., Kaplan A., Caron L., Weber T. (2008). A model for carbohydrate metabolism in the diatom Phaeodactylum tricornutum deduced from comparative whole genome analysis. PLoS ONE.

[60] Zulu N.N., Zienkiewicz K., Vollheyde K., Feussner I. (2018). Current trends to comprehend lipid metabolism in diatoms. Prog. Lipid Res..

[61] Wu Y., Campbell D.A., Irwin A.J., Suggett D.J., Finkel Z.V. (2014). Ocean acidification enhances the growth rate of larger diatoms. Limnol. Oceanogr..

[62] Tsuzuki M., Ohnuma E., Sato N., Takaku T., Kawaguchi A. (1990). Effects of CO2 concentration during growth on fatty acid composition in microalgae. Plant Physiol..

[63] Sato N. (1989). Modulation of lipid and fatty acid content by carbon dioxide in Chlamydomonas reinhardtii. Plant Sci..

[64] Suffrian K., Schulz K.G., Gutowska M., Riebesell U., Bleich M. (2011). Cellular pH measurements in Emiliania huxleyi reveal pronounced membrane proton permeability. New Phytol..

[65] Rossoll D., Bermúdez R., Hauss H., Schulz K.G., Riebesell U., Sommer U., Winder M. (2012). Ocean acidification-induced food quality deterioration constrains trophic transfer. PLoS ONE.

[66] Rousch J.M., Bingham S.E., Sommerfeld M.R. (2003). Changes in fatty acid profiles of thermo-intolerant and thermo-tolerant marine diatoms during temperature stress. J. Exp. Mar. Biol. Ecol..

[67] Van Wagenen J., Miller T.W., Hobbs S., Hook P., Crowe B., Huesemann M. (2012). Effects of light and temperature on fatty acid production in Nannochloropsis salina. Energies.

[68] Sayanova O., Mimouni V., Ulmann L., Morant-Manceau A., Pasquet V., Schoefs B., Napier J.A. (2017). Modulation of lipid biosynthesis by stress in diatoms. Philos. Trans. R. Soc. B Biol. Sci..

[69] Peng K.T., Zheng C.N., Xue J., Chen X.Y., Yang W.D., Liu J.S., Bai W., Li H.Y. (2014). Delta 5 fatty acid desaturase upregulates the synthesis of polyunsaturated fatty acids in the marine diatom Phaeodactylum tricornutum. J. Agric. Food Chem..

[70] Cook O., Hildebrand M. (2015). Enhancing LC-PUFA production in Thalassiosira pseudonana by overexpressing the endogenous fatty acid elongase genes. J. Appl. Phycol..

[71] Ma Y.-H., Wang X., Niu Y.-F., Yang Z.-K., Zhang M.-H., Wang Z.-M., Yang W.-D., Liu J.-S., Li H.-Y. (2014). Antisense knockdown of pyruvate dehydrogenase kinase promotes the neutral lipid accumulation in the diatom Phaeodactylum tricornutum. Microb. Cell Factories.

[72] Trentacoste E.M., Shrestha R.P., Smith S.R., Glé C., Hartmann A.C., Hildebrand M., Gerwick W.H. (2013). Metabolic engineering of lipid catabolism increases microalgal lipid accumulation without compromising growth. Proc. Natl. Acad. Sci. USA.

[73] Sunda W.G., Price N.M., Morel F.M. (2005). Trace metal ion buffers and their use in culture studies. Algal Cult. Tech..

[74] Pachauri R.K., Allen M.R., Barros V.R., Broome J., Cramer W., Christ R., Church J.A., Clarke L., Dahe Q., Dasgupta P. (2014). Climate Change 2014: Synthesis Report. Contribution of Working Groups I, II and III to the Fifth Assessment Report of the Intergovernmental Panel on Climate Change.

[75] Pierrot D., Lewis E., Wallace D. (2006). CO2SYS DOS Program Developed for CO2 System Calculations.

[76] Gao G., Gao K., Giordano M. (2009). Responses to solar radiation of the diatom Skeletonema coastatum (Bacillariphyceae) grown at different Zn(2+) concentrations (1). J. Phycol..

[77] Bradford M.M. (1976). A rapid and sensitive method for the quantitation of microgram quantities of protein utilizing the principle of protein-dye binding. Anal. Biochem..

[78] Deriaz R. (1961). Routine analysis of carbohydrates and lignin in herbage. J. Sci. Food Agric..

[79] Folch J., Lees M., Stanley G.S. (1957). A simple method for the isolation and purification of total lipides from animal tissues. J. Biol. Chem..

[80] Bolger A.M., Lohse M., Usadel B. (2014). Trimmomatic: A flexible trimmer for Illumina sequence data. Bioinformatics.

[81] Grabherr M.G., Haas B.J., Yassour M., Levin J.Z., Thompson D.A., Amit I., Adiconis X., Fan L., Raychowdhury R., Zeng Q. (2011). Full-length transcriptome assembly from RNA-Seq data without a reference genome. Nat. Biotechnol..

[82] Waterhouse R.M., Seppey M., Simao F.A., Manni M., Ioannidis P., Klioutchnikov G., Kriventseva E.V., Zdobnov E.M. (2018). BUSCO Applications from Quality Assessments to Gene Prediction and Phylogenomics. Mol. Biol. Evol..

[83] Mitchell A.L., Attwood T.K., Babbitt P.C., Blum M., Bork P., Bridge A., Brown S.D., Chang H.Y., El-Gebali S., Fraser M.I. (2019). InterPro in 2019: Improving coverage, classification and access to protein sequence annotations. Nucleic Acids Res..

[84] Pruitt K.D., Tatusova T., Maglott D.R. (2007). NCBI reference sequences (RefSeq): A curated non-redundant sequence database of genomes, transcripts and proteins. Nucleic Acids Res..

[85] El-Gebali S., Mistry J., Bateman A., Eddy S.R., Luciani A., Potter S.C., Qureshi M., Richardson L.J., Salazar G.A., Smart A. (2019). The Pfam protein families database in 2019. Nucleic Acids Res..

[86] Kanehisa M., Goto S. (2000). KEGG: Kyoto encyclopedia of genes and genomes. Nucleic Acids Res..

[87] Leng N., Dawson J.A., Thomson J.A., Ruotti V., Rissman A.I., Smits B.M., Haag J.D., Gould M.N., Stewart R.M., Kendziorski C. (2013). EBSeq: An empirical Bayes hierarchical model for inference in RNA-seq experiments. Bioinformatics.

[88] Tarazona S., Garcia-Alcalde F., Dopazo J., Ferrer A., Conesa A. (2011). Differential expression in RNA-seq: A matter of depth. Genome Res..

[89] Love M.I., Huber W., Anders S. (2014). Moderated estimation of fold change and dispersion for RNA-seq data with DESeq2. Genome Biol..

[90] Audic S., Claverie J.-M. (1997). The significance of digital gene expression profiles. Genome Res..

