# Supplementary material for: The Biotechnological Potential of the Marine Diatom Skeletonema dohrnii to the Elevated Temperature and pCO2
Source: Mar Drugs. 2020 May 15;18(5):259. doi: 10.3390/md18050259 (PMC7281586; doi:10.3390/md18050259)
Supplement: Supplementary file 1 [file marinedrugs-18-00259-s001.zip › marinedrugs-749528-supplementary/Supplementary-Figures.pdf]

Supplementary Figures

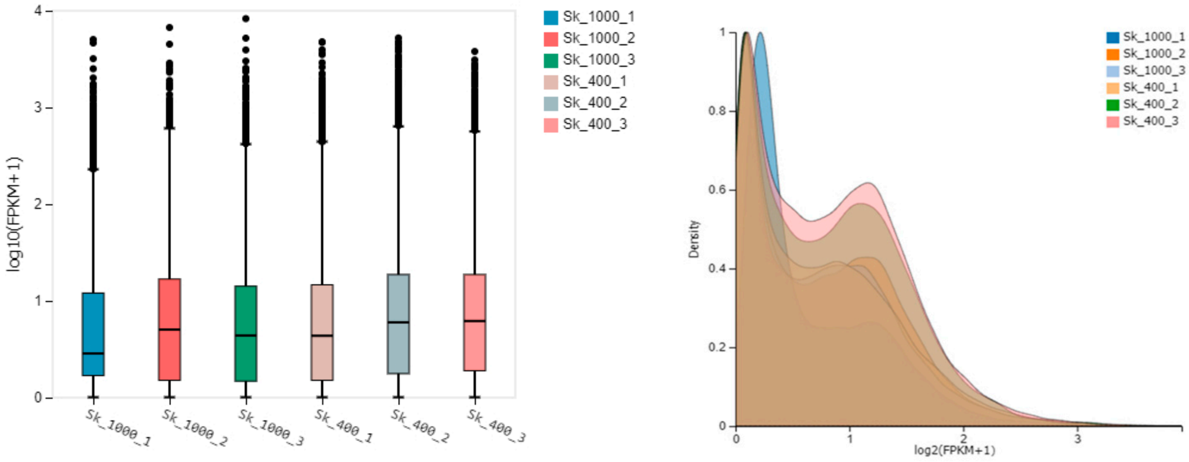

Figure S1. Unigenes FPKM distribution and density variation among six samples. Please see (Table S1) for the comprehensive FPKM distribution of each sample. The box plot shows the distribution of gene expression levels of each sample, and the degree of dispersion of the data distribution can be observed. The density map shows trend of each gene abundance in the sample with the expression level and can clearly reflect the interval in which the gene expression level in the sample is concentrated. The X axis is the sample and the Y axis is log10 (FPKM +1). The box plot of each area corresponds to five statistics (from top to bottom are upper limit, upper quartile, median, lower quartile Number of digits, lower limit, where the upper and lower limits do not consider outliers). The X axis is log2 (FPKM +1), and the Y axis is the density of genes, that is the ratio of the number of genes at this expression level to the total number of expressed genes.

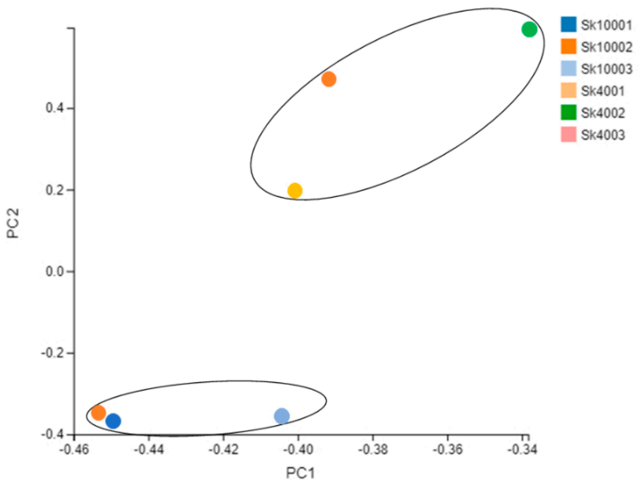

Figure S2. Principal component analysis of the expressed genes in two different CO_2 concentrations

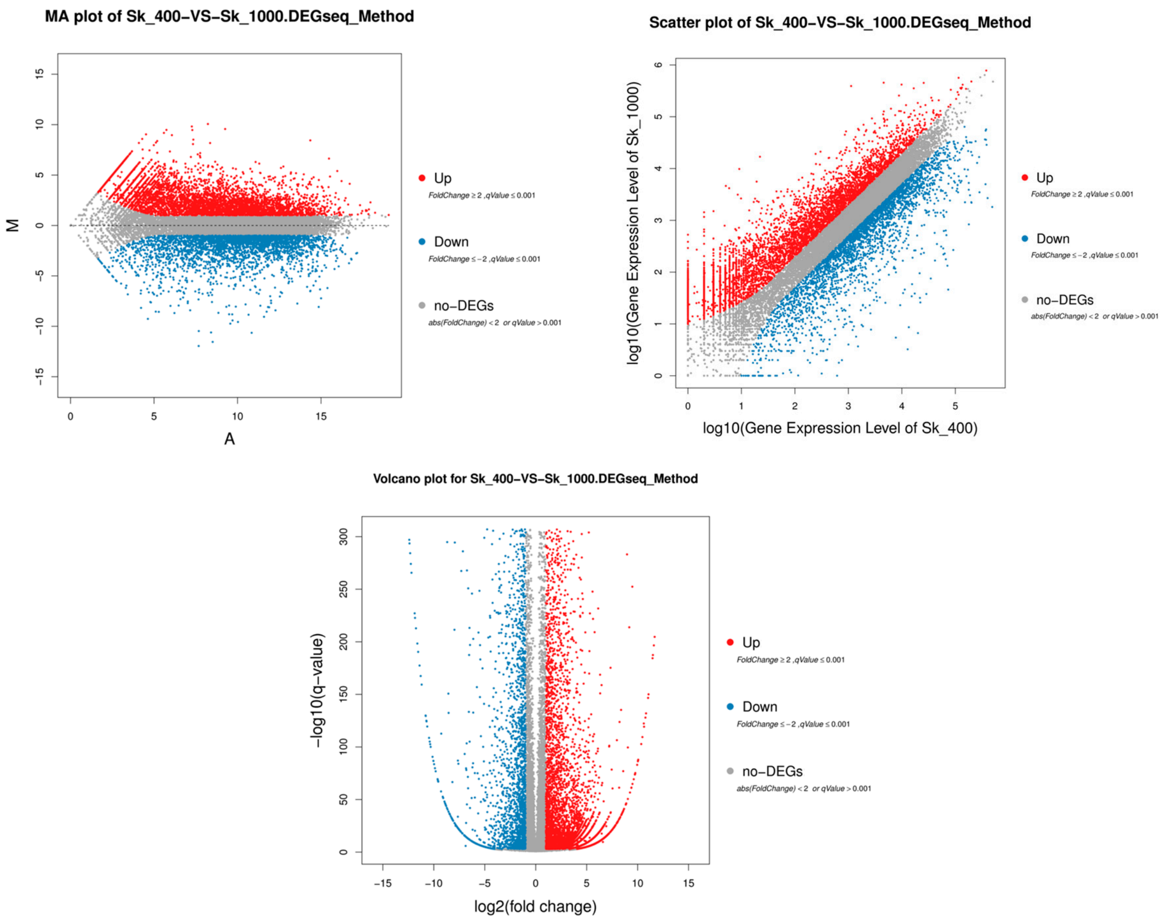

Figure S3. MA, Scatter and volcano plot summarizing differentially expressed RNA-Sequence data for high and low $p\mathrm{CO_2}$ comparison.

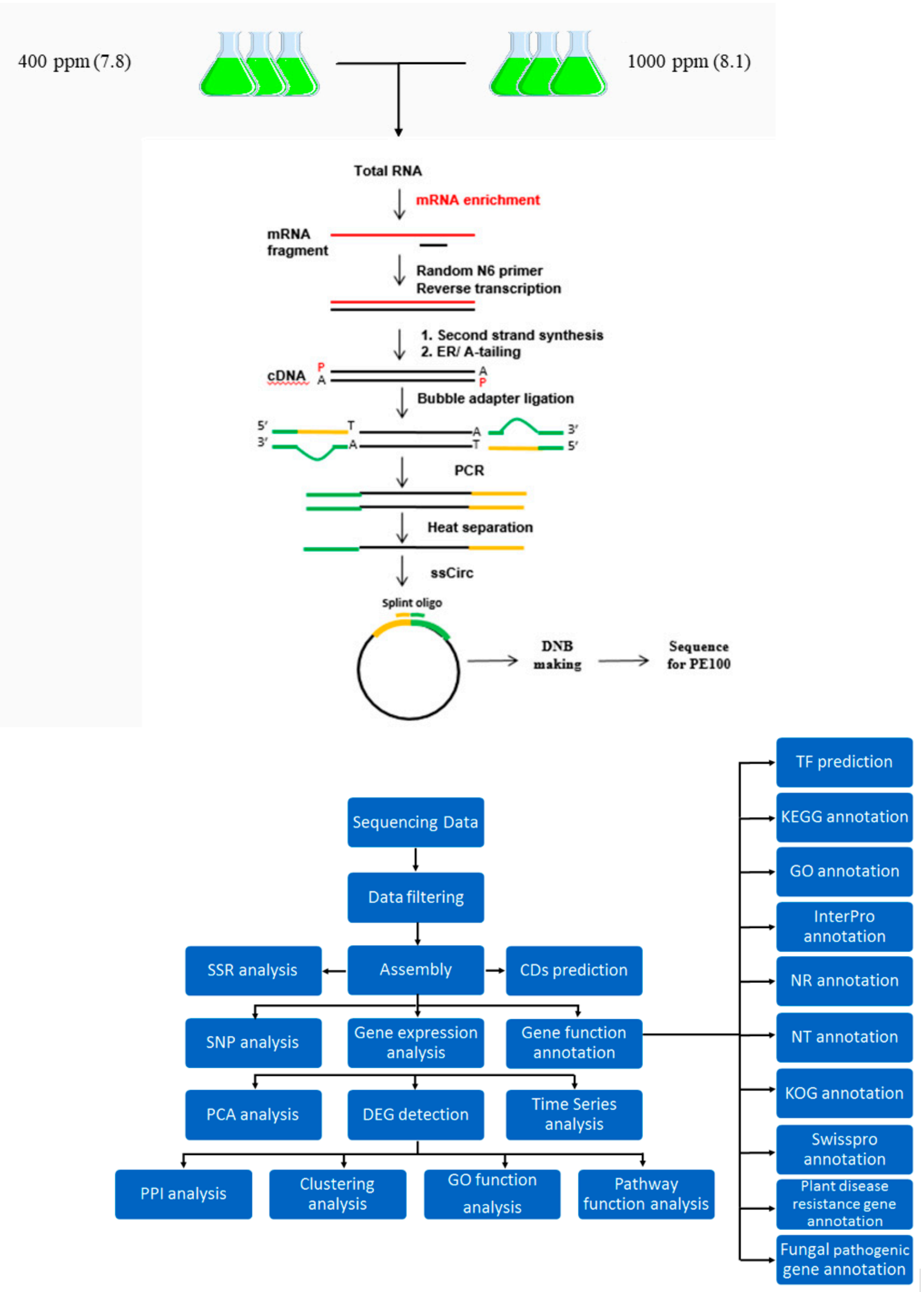

Figure S4. Experimental setup and carried out bioinformatics pipeline.
